# The role of inflammatory cytokines and blood metabolites in osteoporosis: A Mendelian randomization study on pathways and therapeutic targets

**DOI:** 10.1097/MD.0000000000046664

**Published:** 2025-12-12

**Authors:** Yuxi Liu, Daxiong Feng, Huajian Jiang, Fabing Tang, Liming Wu, Songquan Mo, Jianhong Tao

**Affiliations:** a Department of Orthopaedics, Santai People’s Hospital, Mianyang, Sichuan Province, China; b Department of Orthopaedics, The Affiliated Hospital of Southwest Medical University, Luzhou, Sichuan Province, China.

**Keywords:** cytokines, inflammation, Mendelian randomization analysis, metabolomics, osteoporosis

## Abstract

The precise mechanisms underlying the development of osteoporosis (OP) remain unclear, but evidence suggests that inflammatory cytokines and blood metabolites play a significant role. We aim to investigate, as a strictly genomic study based on genome-wide association studies (GWAS) summary statistics, the causal relationships between inflammatory cytokines, blood metabolites, and OP by using Mendelian randomization (MR) analysis and uncover potential therapeutic targets. We utilized inflammatory cytokines from a GWAS summary containing 8293 healthy participants, 1400 blood metabolites from GWAS Catalog, and OP data from the FinnGen repository, all of which are sourced from the largest GWAS conducted to date. Employing bidirectional MR analyses, we investigated the causal relationships between inflammatory cytokines and OP. Additionally, we conducted 2 mediation analyses, two-step MR and multivariable MR (MVMR), to identify potential mediating metabolites. Three inflammatory cytokines were causally associated with OP, while OP did not have a significant causal effect on them. In the two-step MR analysis, CTACK and TRAIL, along with metabolites Spermidine to (N(1) + N(8))-acetylspermidine ratio, Oxalate, 2,4-di-tert-butylphenol, Phosphate to linoleoyl-arachidonoyl-glycerol (18:2–20:4) ratio, Trans 3,4-methyleneheptanoate and X-11478, were all significantly associated with OP (all *P* < .05). MVMR analysis revealed that the associations between CTACK and OP were mediated by Spermidine to (N(1) + N(8))-acetylspermidine ratio (−15.6%, *P* = .001), Oxalate (7.8%, *P* = .014), and 2, 4-di-tert-butylphenol (−15.6%, *P* = .005). The present MR study offers evidence supporting the causal relationships between several specific inflammatory cytokines and OP, as well as identifying potential mediating metabolites.

## 1. Introduction

Osteoporosis (OP) is a chronic metabolic disorder affecting the entire body, marked by reduced bone mineral density (BMD) and a higher risk of fractures.^[1]^ In recent years, the increasing aging population has been a key factor in the growing prevalence of OP and osteoporotic fractures, which has imposed a substantial burden on healthcare systems and negatively impacted quality of life.^[2,3]^ Investigating the underlying mechanisms of OP and developing novel therapeutic approaches remain essential for tackling the urgent challenge of its prevention and treatment. OP shows a marked sex difference, disproportionately affecting postmenopausal women due to estrogen deficiency and related risk factors, and globally an estimated one in 3 women and one in 5 men over age 50 will sustain an osteoporotic fracture, underscoring the greater burden in women.^[4,5]^

Inflammatory cytokines are small, bioactive proteins that regulate inflammation and immune responses and are predominantly produced and released by immune cells.^[6]^ Research has established a clear link between elevated inflammatory cytokines and OP, suggesting that inhibiting their activity may help mitigate OP caused by chronic inflammation.^[7]^ Metabolites, which are intermediate or end products of metabolic processes, are small molecules that fluctuate in response to various factors, including inflammatory cytokines. These metabolites not only have the potential to influence disease risk but also serve as crucial targets for therapeutic interventions. For example, Nicotinamide and NAD + boosting precursors are being explored as metabolic therapeutic targets for OP: in aging mice, nicotinamide riboside increased NAD + in osteoblast-lineage cells and mitigated bone loss. Consistently, nicotinamide mononucleotide reduced osteoporotic changes and enhanced bone healing in ovariectomized mice, highlighting NAD + metabolism as an actionable target for future clinical evaluation.^[8,9]^ Previous studies have also explored the relationship between blood metabolites and BMD. For instance, one study identified 10 blood metabolites that may affect femoral neck BMD, while a more recent study revealed 13 plasma metabolites that significantly impact heel BMD.^[10,11]^ Thus, combining blood metabolite analysis with inflammatory cytokine research could be crucial in discovering biomarkers and targeted therapies for OP.

Mendelian randomization (MR) is a powerful method used to identify potential causal relationships between exposure and outcome.^[12]^ It leverages genetic variations associated with exposure as instrumental variables (IVs) to assess their causal effects.^[13]^ This approach allows MR to be used as a tool for inferring causal relationships between inflammatory cytokines and OP. In our study, we performed bidirectional and mediation MR analyses based on summary statistics from the largest and most recent genome-wide association studies (GWAS) of inflammatory cytokines, blood metabolites, and OP. This allowed us to comprehensively dissect the associations among these factors.

## 2. Methods

### 2.1. Study design and data sources

**Figure 1.**
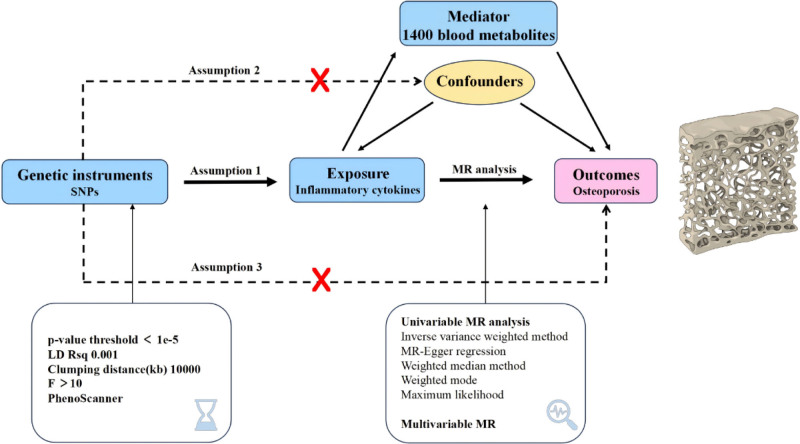
Assumptions and design of the bidirectional and mediation Mendelian randomization (MR) analyses. Initially, a two-sample bidirectional MR analysis was conducted to explore the causality between inflammatory cytokines and osteoporosis (OP). Subsequently, a two-step MR approach was utilized to identify potential mediators (Step 1: the impact of metabolites on OP; Step 2: the influence of inflammatory cytokines on metabolites), which was then validated through multivariable MR.

Figure 1 illustrates the study design, underscoring that the causal interpretations derived from MR estimates are contingent upon 3 fundamental assumptions. Specifically, the genetic variants, serving as IVs or single nucleotide polymorphisms (SNPs), are required to strongly predict the exposures, be associated with the outcome exclusively through these exposures, and not be connected to any confounders that could affect the exposure-outcome relationship.^[13]^ Ethical approval for each GWAS included in this study can be accessed through the respective original articles.

The GWAS data for 41 inflammatory cytokines were sourced from a study that identified genetic variant associations with these cytokines and growth factors in a cohort of 8293 Finnish individuals.^[14]^ Summary statistics for 1400 blood metabolites were obtained from the GWAS Catalog (https://www.ebi.ac.uk/gwas/), representing an unparalleled resource of genetic associations.^[10]^ The outcome data were sourced from the FinnGen Consortium’s R12 release, an extensive genomic database with detailed phenotypic information. For this study, we specifically focused on data labeled as “OP” within this dataset. Selecting this specific label was crucial to ensure an accurate and relevant definition of OP for our study.^[15]^

### 2.2. Instrumental variable (IV)

To select IVs, the following quality control procedures were implemented: First, SNPs with a *P*-value below the locus-wide significance threshold (1 × 10^−5^) were identified as IVs strongly linked to the inflammatory cytokines and blood metabolites. For reverse analysis, SNPs associated with OP meeting the conventional GWAS threshold (*P* < 5 × 10^−8^) were selected. Second, to ensure IV independence, linkage disequilibrium among SNPs was assessed using clumping (*R*^2^ < 0.001, clumping distance = 10,000 kb). Third, screened SNPs were harmonized with the outcome’s GWAS summary statistics, excluding palindromic and ambiguous alleles. Fourth, the *F*-statistic of each SNP was calculated to assess its statistical strength, discarding those with *F*-statistics < 10 to mitigate weak IV bias.^[16]^ Fifth, SNPs in the harmonized dataset strongly linked to the outcome (*P* < 1 × 10^−5^) were manually reviewed and excluded. Sixth, the MR–PRESSO test was utilized to assess potential horizontal pleiotropy and identify outliers. The refined list of SNPs, post-outlier elimination, was then utilized for further MR analysis.

Applying these criteria, we obtained 598 SNPs for 41 cytokines, 28,512 SNPs for 1400 metabolites, and 109 SNPs for OP. All IVs exhibited *F*-values >10, indicating their robustness in minimizing bias introduced by weak IVs. Details about the selected IVs are provided in Tables S1–S3 (Supplemental Digital Content, https://links.lww.com/MD/Q971).

### 2.3. Two-sample bidirectional MR

We first conducted two-sample bidirectional MR analyses to explore the causal relationship between the inflammatory cytokines and OP. For effect estimates, we used the conventional MR approach: the inverse-variance-weighted (IVW) method. Results were reported as beta (β) values with standard errors for continuous outcomes, and odds ratios (ORs) with 95% confidence intervals (CIs) for binary outcomes; *P*-values below 0.05 were considered nominally significant. In addition, we performed 4 complementary methods: MR Egger, the weighted median method, the weighted mode method, and maximum likelihood.

### 2.4. Two-step MR and MVMR

We further employed 2 mediation approaches, two-step Mendelian randomization (TSMR) and multivariable Mendelian randomization (MVMR),^[17,18]^ to analyze both the direct and indirect effects of inflammatory cytokines and 1400 blood metabolites on OP. TSMR assumes that there is no interaction between the exposure and the mediator. In addition to the basic effect estimates of inflammatory cytokines on OP (β) derived from univariate MR analyses, 2 additional estimates were calculated: the causal effect of the mediator (1400 blood metabolites) on OP (β2), and the causal effect of the exposure (the 4 inflammatory cytokines identified in the primary MR analysis) on the mediator (β3).

Finally, we used MVMR as an additional method to validate the roles of metabolites identified in TSMR. MVMR estimates the controlled direct effect of the exposure on the outcome. In our study, this includes the effect of metabolites on OP, adjusting for inflammatory cytokines (β2*), and the effect of inflammatory cytokines on OP, adjusting for metabolites (β1*).^[19]^ The indirect effect, representing the causal effect of inflammatory cytokines on OP via mediators, can be estimated using the product of coefficients method (β3 × β2*). Consequently, the proportion mediated can be calculated as the “indirect effect divided by the total effect” ([β3 × β2*]/β). Our analytic process followed the STROBE-MR guidelines.^[20]^

### 2.5. Statistical analysis

We conducted sensitivity analyses using the MR-Egger regression, leave-one-out, and MR-PRESSO methods. The MR-Egger regression and MR-PRESSO were utilized to test for and correct potential horizontal pleiotropy in our selected IVs. MR-Egger regression was employed to account for horizontal pleiotropy by relying on the InSIDE (Instrument Strength Independent of Direct Effect) assumption, which ensures the strength of genetic variants’ effect on the exposure is independent of their effect on the outcome.^[21]^ MR-Egger estimates both the causal effect (slope) and pleiotropy (intercept), with a non-zero intercept indicating pleiotropic bias. The *P*-value from the MR-Egger regression intercept test was applied to evaluate horizontal pleiotropy, and the method provided bias-adjusted causal estimates and robustness assessments. Additionally, MR-PRESSO was used to detect and eliminate outliers among the IVs. MR-PRESSO includes 3 components: the Global Test to identify overall pleiotropy, the Outlier Test to flag and remove pleiotropic IVs, and the Distortion Test to assess the impact of outlier correction on causal estimates.^[22]^ By systematically addressing pleiotropy, MR-PRESSO enhanced the reliability of our causal estimates. Furthermore, Cochrane *Q* statistic was applied to assess the variability of SNP estimates across each MR association, and MVMR analysis was implemented to adjust for confounding risk factors, effectively minimizing their influence on the causal inference. The statistical methods employed in our study have been thoroughly reviewed and rigorously validated by a statistician to ensure their accuracy and reliability.

To control for multiple comparisons in our analysis, we applied the false discovery rate (FDR) correction using the *q*-value approach. The *q*-value provides an estimate of the proportion of false positives among the set of significant results, allowing for a balance between statistical power and the control of false discoveries. This method was chosen due to its suitability for large-scale genomic data and its ability to provide interpretable significance thresholds.^[23]^ All IVW results were adjusted for multiple testing using the FDR method, with a significance threshold set at *q*-value < 0.1. Results indicating a *P*-value < .05 but a *q*-value ≥ 0.1 were considered suggestive of an association. All MR analyses were conducted in R (version 4.3.2; R Foundation for Statistical Computing, Vienna, Austria), utilizing the “TwoSampleMR” and “MendelianRandomization” packages. FDR *q*-values were estimated using the “p.adjust” function in R.

## 3. Results

### 3.1. Causal effects of inflammatory cytokines on OP

**Figure 2.**
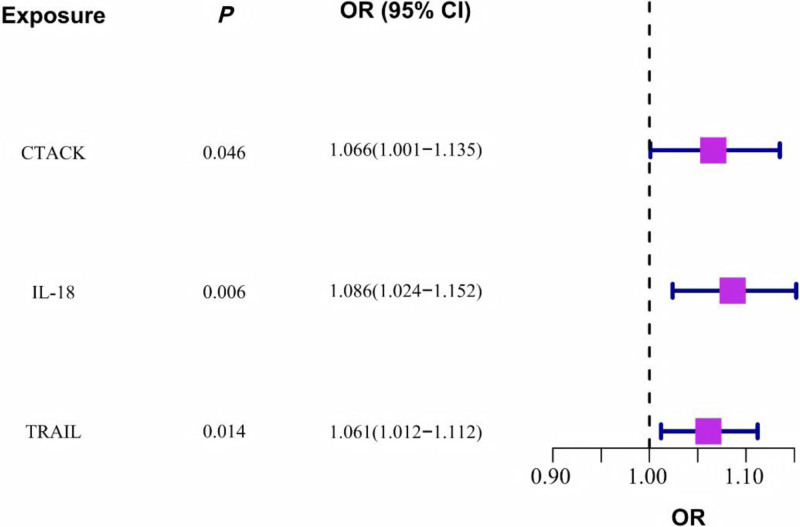
Mendelian randomization analyses demonstrate the causal effect of inflammatory cytokines on osteoporosis. The purple squares indicate positive odds ratios (ORs) derived from the inverse-variance-weighted (IVW) analysis (truncated at a *P*-value of < .05). CI = confidence interval.

When evaluating the causal effects of inflammatory cytokines on OP, using the IVW method, 3 cytokines were positively associated with OP (Fig. 2 and Table S4, Supplemental Digital Content, https://links.lww.com/MD/Q971). Among these 3 cytokines, IL-18 had the strongest causal effect on increasing the risk of OP (OR = 1.086, 95% CI = 1.024–1.152, *P* = .006). In the reverse MR analysis, no significant causal effect of OP on inflammatory cytokines was observed (Table 1). These results are considered reliable: sensitivity analyses showed no evidence of horizontal pleiotropy or outliers (MR-Egger intercept; MR-PRESSO), low heterogeneity (Cochran *Q*), stable leave-one-out profiles, and instrument strength exceeding the conventional *F* > 10 threshold (Tables S5 and S6, Supplemental Digital Content, https://links.lww.com/MD/Q971).

**Table 1:** Mendelian randomization analyses demonstrating the causal effects of osteoporosis on inflammatory cytokines.

Exposure	Outcome	Method	Number of SNP	OR (95% CI)	*P*	*q*-value
Osteoporosis	CTACK	IVW	36	1.043 (0.876 ± 1.240)	.638	0.638
		MR Egger	36	1.423 (0.641 ± 3.160)	.392	
		Weighted median	36	1.032 (0.819 ± 1.301)	.788	
		Weighted mode	36	1.058 (0.686 ± 1.631)	.801	
		ML	36	1.044 (0.886 ± 1.230)	.608	
	IL-18	IVW	36	1.139 (0.969 ± 1.339)	.115	0.345
		MR Egger	36	1.057 (0.504 ± 2.217)	.883	
		Weighted median	36	1.175 (0.929 ± 1.486)	.178	
		Weighted mode	36	1.151 (0.658 ± 2.014)	.626	
		ML	36	1.143 (0.970 ± 1.346)	.110	
	TRAIL	IVW	37	1.043 (0.932 ± 1.168)	.464	0.638
		MR Egger	37	0.946 (0.560 ± 1.599)	.838	
		Weighted median	37	1.039 (0.888 ± 1.217)	.632	
		Weighted mode	37	1.131 (0.794 ± 1.611)	.500	
		ML	37	1.044 (0.937 ± 1.164)	.433	

OR, 95% CI, and *P*-values were derived from Mendelian randomization analysis, while *q*-values were calculated using the false discovery rate method.

CI = confidence interval, IVW = inverse-variance-weighted, ML = maximum likelihood, MR = Mendelian randomization, OR = odds ratio, SNP = single nucleotide polymorphism.

### 3.2. Mediation analyses of 1400 blood metabolites

**Figure 3.**
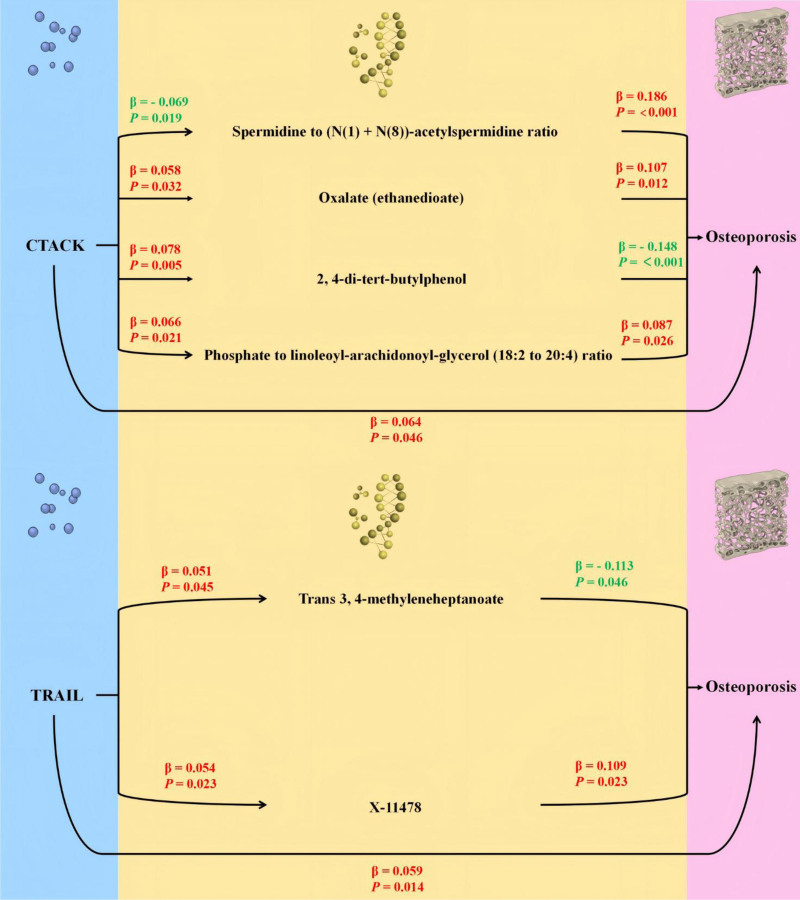
The diagram illustrates the mediation pathway of “inflammatory cytokines – blood metabolites – osteoporosis” in a two-step Mendelian randomization. Beta values (β) represent the causal effect estimates obtained through the inverse-variance-weighted method (truncated at *P* < .05). Characters highlighted in red and green denote positive and negative associations, respectively.

In the TSMR analysis (Fig. 3), it was found that a total of fifty-six blood metabolites were causally associated with OP (Table S7, Supplemental Digital Content, https://links.lww.com/MD/Q971). In the sensitivity analysis, the MR-PRESSO test identified 4 SNPs with pleiotropy (rs138783171, rs71517736, rs10107182, rs3765340). After removing these SNPs, we found that the causal relationship between one of the blood metabolites and OP changed (*P*-value > .05). In the leave-one-out analysis, we identified one pleiotropic IV (rs12658815). After removing this IV, the causal relationship between the corresponding blood metabolite and OP also changed (*P*-value > .05). Additionally, although there were 5 blood metabolites causally associated with OP, the MR-Egger method showed opposite trends, suggesting that the causal relationship may not hold.^[21]^ Among the remaining forty-nine blood metabolites, after multiple testing correction, the IVW result for the Adenosine 5’-monophosphate (AMP) to urate ratio (FDR *q*-value = 0.011) was statistically significant. Additionally, the levels of 3β-hydroxy-5-cholenoic acid (FDR *q*-value = 0.043), Spermidine to (N(1) + N(8))-acetylspermidine ratio (FDR *q*-value = 0.046), Nicotinamide (FDR *q*-value = 0.046), and 2,4-di-tert-butylphenol (FDR *q*-value = 0.056) also reached statistical significance. Although the Cochrane *Q* test indicated heterogeneity for one metabolite (*P*-value < .05) (Table S5, Supplemental Digital Content, https://links.lww.com/MD/Q971), this heterogeneity did not affect the overall result. The presence of heterogeneity may be attributed to the data coming from different analytical platforms or experiments, etc.

Among the 3 inflammatory cytokines causally associated with OP, 2 were significantly correlated with 6 of the forty-nine metabolites mentioned above, as shown in Table 2 and Table S8 (Supplemental Digital Content, https://links.lww.com/MD/Q971). CTACK (OR = 1.066, 95% CI = 1.001–1.135, *P* = .046) and TRAIL (OR = 1.061, 95% CI = 1.012–1.112, *P* = .014) were identified as detrimental inflammatory cytokines associated with OP. Specifically, CTACK exerted its detrimental effects in OP by upregulating the levels of Oxalate, 2,4-di-tert-butylphenol, and the Phosphate to linoleoyl-arachidonoyl-glycerol (18:2–20:4) ratio, and by downregulating the Spermidine to (N(1) + N(8))-acetylspermidine ratio. Similarly, TRAIL demonstrated detrimental effects by upregulating the levels of Trans 3,4-methyleneheptanoate and X-11478.

**Table 2:** Mendelian randomization analyses demonstrating the causal effects of inflammatory cytokines on blood metabolites.

Exposure	Mediator	Method	Number of SNP	Beta ± SE	*P*	*P*_h_	*P*_intercept_
CTACK	Spermidine to (N(1) + N(8))-acetylspermidine ratio	IVW	15	−0.069 ± 0.029	.019	.542	.901
		MR Egger	15	−0.064 ± 0.048	.204	.464	
	Oxalate	IVW	15	0.058 ± 0.027	.032	.952	.543
		MR Egger	15	0.036 ± 0.044	.428	.942	
	2,4-Di-tert-butylphenol	IVW	15	0.078 ± 0.027	.005	.994	.867
		MR Egger	15	0.072 ± 0.045	.134	.989	
	Phosphate to linoleoyl-arachidonoyl-glycerol ratio	IVW	15	0.066 ± 0.029	.021	.670	.377
		MR Egger	15	0.100 ± 0.047	.053	.664	
TRAIL	Trans 3,4-methyleneheptanoate	IVW	21	0.051 ± 0.025	.045	.601	.480
		MR Egger	21	0.036 ± 0.032	.269	.572	
	X-11478	IVW	21	0.054 ± 0.024	.023	.442	.367
		MR Egger	21	0.037 ± 0.030	.238	.433	

Beta, standard errors (SE), and *P*-values were derived from the Mendelian randomization analysis. Heterogeneity testing in the IVW method was conducted using Cochran *Q* statistic.

IVW = inverse-variance-weighted, MR = Mendelian randomization, *P*_*h*_ = *P*-value for heterogeneity, *P*_intercept_ = *P*-value for the intercept of the MR-Egger regression, SNP = single nucleotide polymorphism.

### 3.3. MVMR validation of mediators

Because TSMR serves as a screening approach that assumes no interaction between the exposure and the mediator and cannot account for correlated exposures, we proceeded to MVMR to estimate controlled direct effects and to validate whether the TSMR identified metabolites truly mediate the cytokine and OP associations after mutual adjustment (Table 3). Using MVMR, we calculated the mediating effects and their mediated proportions for these metabolites and found that the levels of Oxalate and 2,4-di-tert-butylphenol, as well as the Phosphate to linoleoyl-arachidonoyl-glycerol ratio, remained significant after adjusting for inflammatory cytokines. For CTACK, the levels of Oxalate and 2,4-di-tert-butylphenol, as well as the Phosphate to linoleoyl-arachidonoyl-glycerol ratio, exhibited mediating effects in the relationship between CTACK and OP, with mediated proportions of 7.8% (*P* = .014), −15.6% (*P* = .005), and −15.6% (*P* = .001), respectively. The mediating effects of Trans 3,4-methyleneheptanoate, X-11478, and the Phosphate to linoleoyl-arachidonoyl-glycerol ratio were insignificant after adjusting for the inflammatory cytokine.

**Table 3:** Multivariable Mendelian randomization analyses of the causal effects of inflammatory cytokines and blood metabolites on osteoporosis.

Exposure	Mediator	Direct effect (β1* ± SE)	Direct effect (β2* ± SE)	*P*	Indirect effect (β3 × β2* ± SE)	Proportion mediated (β3 × β2*/β)
CTACK	Spermidine to (N(1) + N(8))-acetylspermidine ratio	0.057 ± 0.035	0.142 ± 0.044	.001	−0.010 ± 0.005	−0.156
	Oxalate	0.066 ± 0.030	0.089 ± 0.036	.014	0.005 ± 0.003	0.078
	2,4-Di-tert-butylphenol	0.067 ± 0.030	−0.127 ± 0.045	.005	−0.010 ± 0.005	−0.156
	Phosphate to linoleoyl-arachidonoyl-glycerol ratio	0.076 ± 0.035	0.062 ± 0.036	.088	0.004 ± 0.003	0.063
TRAIL	Trans 3,4-methyleneheptanoate	−0.015 ± 0.023	−0.075 ± 0.053	.154	−0.004 ± 0.003	−0.063
	X-11478	−0.014 ± 0.023	0.061 ± 0.048	.206	0.003 ± 0.003	0.047

Beta (β1* and β2*), standard errors (SE), and *P*-values were derived from multivariable Mendelian randomization analysis. β1* and β2* represent the controlled direct effects of each pair of inflammatory cytokines and metabolite on osteoporosis (OP) after adjusting for each other. β3 denotes the causal effect of exposure on the mediator; the indirect effect (β3 × β2*) signifies the effect of exposure on OP via the corresponding mediator; β denotes the total effect of exposure on OP; proportion mediated is calculated as the “indirect effect/total effect.”

## 4. Discussion

In this large-scale MR study, we found that 3 inflammatory cytokines were causally associated with OP, whereas OP did not exert a significant causal effect on these cytokines. Through a two-step MR analysis, we identified CTACK and TRAIL, as well as several metabolites (e.g., Oxalate, 2,4-di-tert-butylphenol, and the Phosphate to linoleoyl-arachidonoyl-glycerol ratio), which were significantly associated with OP. Specifically, CTACK contributed to OP by increasing Oxalate, 2,4-di-tert-butylphenol, and the Phosphate to linoleoyl-arachidonoyl-glycerol ratio, while decreasing the Spermidine to (N(1) + N(8))-acetylspermidine ratio. Similarly, TRAIL exerted adverse effects by elevating Trans 3,4-methyleneheptanoate and X-11478. Moreover, the IVW results for 2,4-di-tert-butylphenol and the Spermidine to (N(1) + N(8))-acetylspermidine ratio retained statistical significance after correction for multiple testing. Additionally, our MVMR analysis revealed that the association between CTACK and OP was partially mediated by Oxalate, 2,4-di-tert-butylphenol, and the Phosphate to linoleoyl-arachidonoyl-glycerol ratio.

A growing body of research indicates that IL levels are strongly linked to OP, with inflammatory cytokines significantly influencing bone metabolism. Among various IL, IL-18 has been recognized as a pivotal factor in OP progression.^[24,25]^ Our study suggests that higher IL-18 levels may contribute to an elevated risk of OP. Supporting our findings, prior research has established a robust connection between IL-18 and pyroptosis. IL-18 mainly activates the NLRP3 inflammasome, which subsequently stimulates osteoclast differentiation, enhances bone resorption, and suppresses osteoblast function.^[26,27]^ Furthermore, a decline in IL-18 binding protein (IL-18BP), a natural inhibitor of IL-18, intensifies its inflammatory effects, worsening bone loss.^[25]^ The dynamic interaction between IL-18, NLRP3 inflammasome activation, and the inflammatory microenvironment underscores its essential role in OP development.^[28,29]^ Targeting IL-18 and its associated signaling pathways – particularly through IL-18BP modulation or NLRP3 inflammasome inhibition – may present innovative therapeutic approaches for OP treatment.^[25,27]^

Our study suggests that CTACK promotes OP progression by inducing metabolic changes, such as increasing Oxalate, 2,4-di-tert-butylphenol, and the Phosphate to linoleoyl-arachidonoyl-glycerol ratio, while decreasing the Spermidine to (N(1) + N(8))-acetylspermidine ratio. These changes likely enhance OP through several mechanisms, including inflammation, impaired osteogenesis, and increased bone resorption. Oxalate accumulation can lead to kidney stones and renal damage by crystal deposition and inflammation, activating the NLRP3 inflammasome and promoting macrophage infiltration, causing chronic inflammation and fibrosis.^[30,31]^ Since bone metabolism is linked to kidney function, Oxalate buildup may indirectly enhance bone loss by increasing systemic inflammation and lowering calcium bioavailability, thus increasing bone resorption and OP risk.^[31,32]^ Moreover, 2,4-di-tert-butylphenol, a synthetic antioxidant, disrupts osteogenic differentiation by downregulating key transcription factors, RUNX2 and OSX, which are essential for osteoblast differentiation and bone formation.^[33]^ Exposure to 2,4-di-tert-butylphenol also reduces calcium deposition, inhibiting bone mineralization and promoting bone resorption. This suggests that CTACK-induced upregulation of 2,4-di-tert-butylphenol impairs osteoblast activity, leading to reduced bone formation and accelerated OP progression. These findings highlight the role of CTACK-induced metabolic dysregulation in OP and suggest its potential as a therapeutic target.

Consistent with the findings of this study, previous research has shown that TRAIL plays an important role in the progression of OP. TRAIL activates the NF-κB and MAPK signaling pathways through TRAF6, thereby promoting the maturation and activation of osteoclasts, the cells responsible for bone resorption.^[34]^ TRAIL also inhibits the bone-protective effects of osteoprotegerin (OPG). OPG typically suppresses osteoclastogenesis by preventing RANKL (Receptor Activator of Nuclear Factor kappa-B Ligand) from binding to RANK receptors on osteoclast precursors, while TRAIL promotes osteoclastogenesis and activity through its interaction with OPG.^[35]^ Other studies have shown that inhibiting TRAIL expression, such as through treatments like Tetrandrine, can reduce osteoclastogenesis and alleviate BMD loss.^[34]^ This study also found that Trans 3,4-methyleneheptanoate and X-11478 mediate the relationship between TRAIL and OP, although the specific mechanism remains unclear and requires further investigation.

To enhance the clinical significance of this study, the findings can be used in OP prevention and personalized treatment by targeting specific cytokines and metabolites. For example, inhibitors of IL-18, CTACK, and TRAIL may help mitigate OP-related damage. Moreover, 2,4-di-tert-butylphenol and the spermidine to (N(1) + N(8))-acetylspermidine ratio represent potential targets for metabolic intervention. These biomarkers support early diagnosis and disease monitoring and enhance treatment efficacy when integrated with cytokine and metabolite-targeted therapies. Thus, this research establishes a basis for precision medicine in OP, promoting more effective and individualized treatments to improve clinical outcomes and slow disease progression.

Despite its contributions, this study has several limitations. First, the MR analysis assumes a linear relationship, which may not fully capture the complexity of biological systems. Non-linear interactions, threshold effects, and the interplay between inflammatory cytokines and blood metabolites could significantly influence OP progression. Future research should integrate non-linear MR models, interaction analyses, and network-based methods to gain deeper insights into these complex dynamics and refine our understanding of causal pathways. Second, since the GWAS data predominantly include individuals of European ancestry, the findings need validation in more diverse populations to improve their reliability and generalizability. Third, sex specific summary GWAS estimates were not consistently available across all traits, and where available the sample sizes were insufficient to ensure instrument strength and statistical power; future studies using larger sex specific GWAS or individual-level data are warranted to evaluate sex differential causal effects. Additionally, we used the FDR correction, which is particularly suitable for large-scale genomic studies with multiple comparisons, as it balances detecting true positives while controlling false discoveries. Unlike the highly conservative Bonferroni correction, which may lower statistical power and overlook subtle associations, FDR preserves sensitivity and provides interpretable significance thresholds.^[36]^ Although some results had marginal *q*-values (e.g., *q* = 0.056) within the FDR-controlled range, independent dataset validation remains crucial. Future studies should replicate these findings in larger, more diverse cohorts and employ innovative analytical approaches to strengthen the evidence.

## Acknowledgments

The authors sincerely thank the investigators who contributed the GWAS summary statistics used in this study.

## Author contributions

**Data curation:** Yuxi Liu, Jianhong Tao.

**Formal analysis:** Yuxi Liu, Jianhong Tao.

**Validation:** Daxiong Feng, Huajian Jiang, Fabing Tang, Liming Wu, Songquan Mo.

**Visualization:** Daxiong Feng, Huajian Jiang, Fabing Tang, Liming Wu, Songquan Mo.

**Writing – original draft:** Yuxi Liu.

**Writing – review & editing:** Daxiong Feng, Huajian Jiang, Fabing Tang, Liming Wu, Songquan Mo, Jianhong Tao.

## Supplementary Material
